# Supplementary figures and images for: Norepinephrine-Induced DNA Damage in Ovarian Cancer Cells
Source: Int J Mol Sci. 2020 Mar 24;21(6):2250. doi: 10.3390/ijms21062250 (PMC7139728; doi:10.3390/ijms21062250)

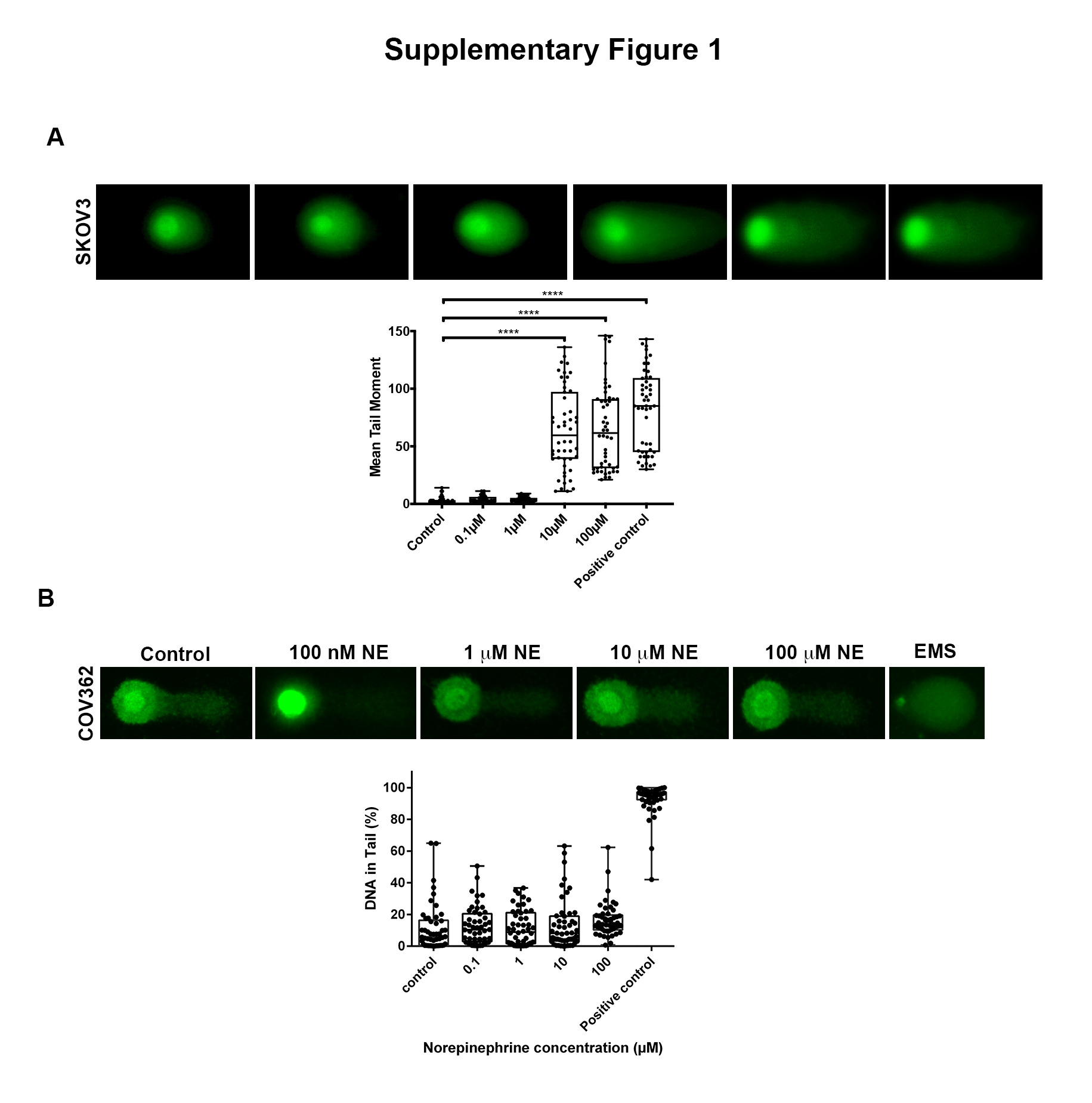

Supplement: Supplementary file 1 [file ijms-21-02250-s001.zip › Supplementary Figure 1_revision2.tif]

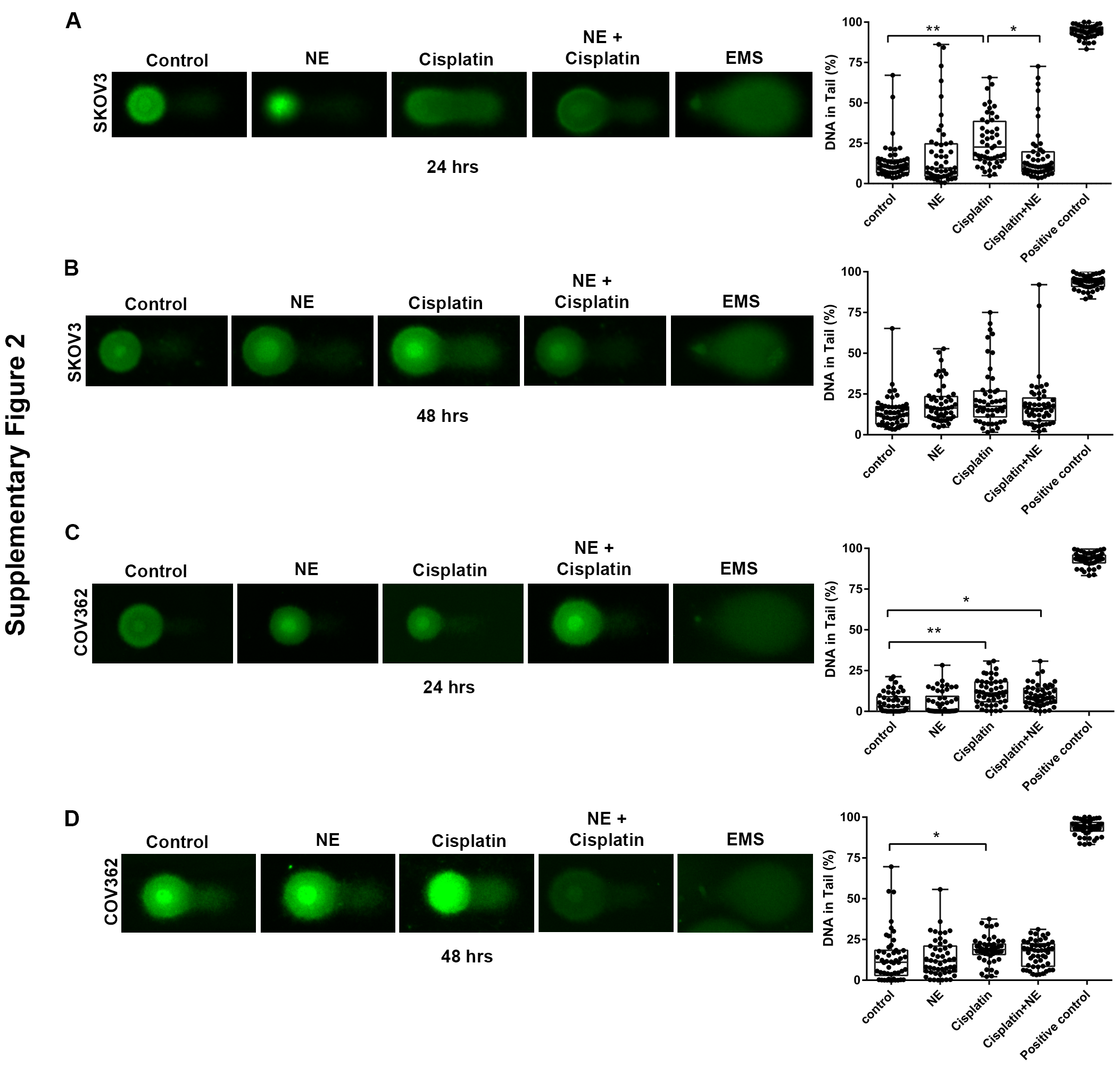

Supplement: Supplementary file 1 [file ijms-21-02250-s001.zip › Supplementary Figure 2_IJMS.tif]
